# Patterning of Metal Halide Perovskite Thin Films and Functional Layers for Optoelectronic Applications

**DOI:** 10.1007/s40820-023-01154-x

**Published:** 2023-07-18

**Authors:** Jin-Wook Lee, Seong Min Kang

**Affiliations:** 1 Department of Nano Engineering and Department of Nano Science and Technology, SKKU Advanced Institute of Nanotechnology (SAINT), Sungkyunkwan University, Suwon, Republic of Korea; 2 SKKU Institute of Energy Science and Technology (SIEST), Sungkyunkwan University, Suwon, Republic of Korea; 3 Department of Mechanical Engineering, Chungnam National University, Daejeon, 34134 Republic of Korea

**Keywords:** Perovskites, Optoelectronics, Light outcoupling, Light harvesting, Patterning

## Abstract

In recent years, metal halide perovskites have received significant attention as materials for next-generation optoelectronic devices owing to their excellent optoelectronic properties. The unprecedented rapid evolution in the device performance has been achieved by gaining an advanced understanding of the composition, crystal growth, and defect engineering of perovskites. As device performances approach their theoretical limits, effective optical management becomes essential for achieving higher efficiency. In this review, we discuss the status and perspectives of nano to micron-scale patterning methods for the optical management of perovskite optoelectronic devices. We initially discuss the importance of effective light harvesting and light outcoupling via optical management. Subsequently, the recent progress in various patterning/texturing techniques applied to perovskite optoelectronic devices is summarized by categorizing them into top-down and bottom-up methods. Finally, we discuss the perspectives of advanced patterning/texturing technologies for the development and commercialization of perovskite optoelectronic devices.

## Highlights

- This review discusses the status and perspectives of nano- to micron-scale patterning method for the optical management of perovskite optoelectronic devices.
- We provide an overview of nanopatterning/texturing technologies for perovskites to achieve a high device performance and categorize them into top-down and bottom-up approaches.

## Graphical abstract

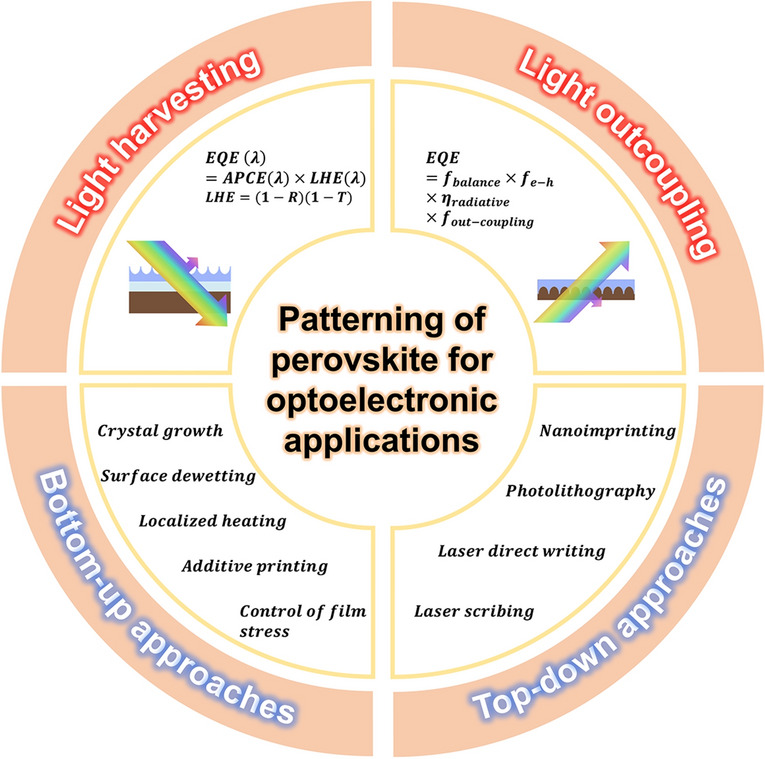

## Introduction

Metal halide perovskites have been established as a new class of inorganic or organic–inorganic hybrid semiconductors featuring excellent optoelectronic properties, such as direct bandgaps, high absorption coefficients, and excellent ambipolar carrier lifetimes and transport capabilities [1]. Owing to their unique optoelectronic properties, numerous studies demonstrating various optoelectronic devices that outperform those based on conventional semiconductors, such as solar cells, light-emitting diodes (LEDs), and X-ray photodetectors, have been conducted [2–5]. In particular, the power conversion efficiency (PCE) of perovskite solar cells (PSCs) has reached 26.0% [6], whereas an external quantum efficiency (EQE) of perovskite light-emitting diodes (PeLEDs) of over 28% has been demonstrated [4]. This impressive performance has motivated companies in relevant industries to consider the commercialization of perovskite optoelectronics.

The significant evolution in the performance of perovskite optoelectronics has been rendered possible by gaining an advanced understanding of the composition, crystal growth, and defect engineering of perovskites [7–10]. Meanwhile, studies on optical management have thus far attracted relatively less attention, probably owing to comparatively less pronounced effects on the device performance. However, as the device performance approaches its thermodynamic limit, the importance of optical management via film and device structuring is critical for achieving a close-to-ideal device performance [11]. In this regard, nano- to micron-scale patterning of metal halide perovskite thin films and other functional layers is an essential technique for optical management in PSCs and PeLEDs. Furthermore, an effective patterning technique can be used to produce solar modules or displays with minimal process costs and enhanced performance.

In this paper, we review important studies on nano- to micron-scale patterning of metal halide perovskite thin films and other functional layers for optoelectronic applications. The importance of light management is first discussed in terms of light harvesting and outcoupling, followed by a review of top-down and bottom-up approaches for patterning. Finally, perspectives toward the practical applications of patterning technologies in high-performance devices are discussed.

## Importance of Light Management by Patterning

### Light Harvesting

Light-harvesting properties are important factors influencing the performance of photovoltaic devices. In solar cells, PCE is directly proportional to the short-circuit photocurrent density (*J*_SC_) of the device, as determined by the following equation [12]:

1 $$J_{{{\text{SC}}}} = \int {q{\text{EQE}} \left( \lambda \right)\phi_{{{\text{ph}},\lambda }}^{{{\text{AM}}1.5G}} d\lambda }$$

where *q* is the elementary charge; ${\text{EQE}} \left( \lambda \right)$ is the wavelength-dependent EQE of the device; and $\phi_{{{\text{ph}},\lambda }}^{{{\text{AM}}1.5G}}$ is the photon flux under AM1.5G standard illumination conditions. ${\text{EQE}} \left( \lambda \right)$ is a function of the light-harvesting efficiency (LHE) and absorbed photon-to-current conversion efficiency (APCE), according to the following equations [13]:

2 $${\text{EQE}} \left( \lambda \right) = {\text{APCE}}\left( \lambda \right) \times {\text{LHE}}\left( \lambda \right)$$

3 $${\text{LHE}} = \left( {1 - R} \right)\left( {1 - T} \right)$$

where *R* is the reflectance, and *T* is the transmittance. Both the APCE and LHE can be improved via nanopatterning or texturing of the perovskite and other functional layers. The incorporation of micro-/nanostructures can be used to control the antireflection effect and the light-trapping and light-scattering mechanisms, which contribute to changes in both *R* and *T*. Meanwhile, the morphology of the perovskite and other functional layers and their interfaces can also affect the APCE, which is determined by the charge collection efficiency.

**Fig. 1:**
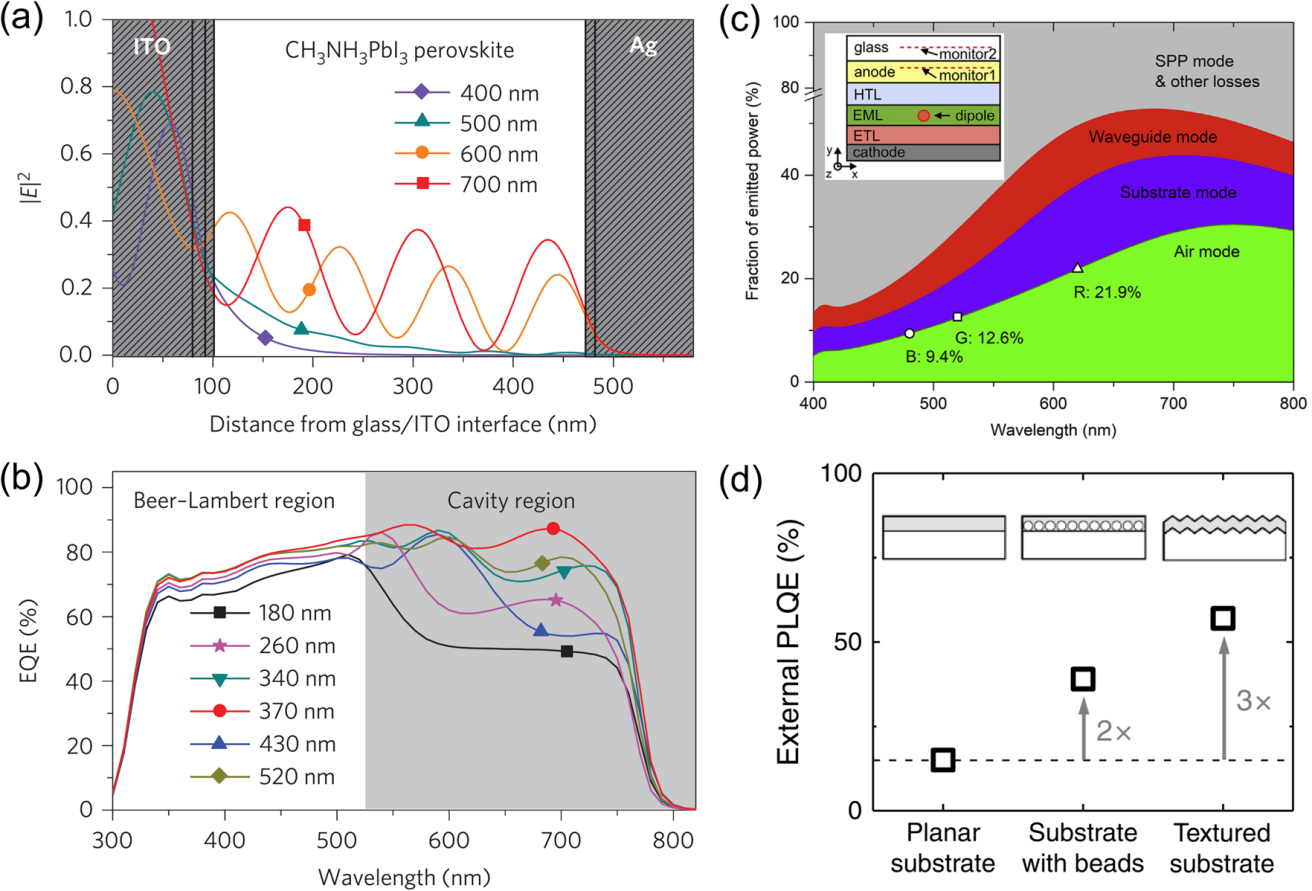
**a** Optical-field distribution in a CH_3_NH_3_PbI_3_-perovskite device for four wavelengths. **b** EQE spectra of devices with different CH_3_NH_3_PbI_3_-layer thicknesses, reproduced from Ref. [15] with permission from Springer Nature. **c** Fractions of various power modes of patterned PeLEDs with an isotropic dipole orientation as a function of the dipole-emitting wavelength. Theoretical maximums of red (620 nm, circle), green (520 nm, star), and blue (480 nm, triangle) emitted powers into air are marked. Inset is the schematic of the device with the use of a light extraction structure, reproduced from Ref. [23] with permission from Elsevier. **d** External PLQEs for MAPbI_3-*x*_Cl_*x*_ on different substrates. Film on a textured substrate exhibits an external PLQE of 57%, which is three times higher than that of the planar film (20%; indicated by the dashed line). Reproduced from Ref. [24] with permission from Springer Nature

State-of-the-art PSCs with very high efficiencies comprise planar heterojunction structures, where a submicron-thick perovskite layer is sandwiched between the charge-transporting layers and electrodes. The light incident to the planar surface of the device is reflected at the heterointerfaces where the refractive index of the medium changes. For example, at the front of the surface, where the light is incident from air to fluorine doped tin oxide (FTO) glass, 5%–10% of the incident light can be reflected depending on its wavelength and incident angle [14]. With a typical perovskite composition with a bandgap of ~ 1.5 eV, such light reflection loss can result in a loss of *J*_SC_ of 1–2 mA cm^−2^, which is significant in terms of the device efficiency. Such reflection loss can occur at every heterointerface in the device, reducing the LHE and thereby the device efficiency. Furthermore, a part of the light incident on the perovskite layer cannot be fully absorbed when the aforementioned layer is not sufficiently thick. In particular, considering the absorption coefficient of perovskite materials, light with a wavelength close to the bandgap cannot be fully absorbed by the submicron-thick perovskite layer (typically > 550 nm for iodide-based perovskites with a bandgap of ~ 1.5 eV), and consequently, the optical field reaches the back electrode (typically metal) to induce optical interference (Fig. 1a, b) [15]. As a result, the EQE in this design can be strongly affected by the microstructure of the perovskite and other functional layers [16]. Therefore, optical management via micro/nanopatterning in PSCs plays a crucial role in the performance of PSCs.

### Light Outcoupling

The EQE of the LED devices represents the conversion efficiency of the injected charge carriers to be converted into emitted photons at a given wavelength. The EQE of the electroluminescence (EL) for an LED is described by the following equation:

4 $${\text{EQE}} = f_{{{\text{balance}}}} \times f_{e - h} \times \eta_{{{\text{radiative}}}} \times f_{{\text{out-coupling}}}$$

where $f_{{{\text{balance}}}}$ is the probability of balanced charge injection; $f_{e - h}$ is the probability of forming correlated electron–hole pairs or excitons from each pair of injected carriers; $\eta_{{{\text{radiative}}}}$ is the probability of radiative recombination for each electron–hole pair; and $f_{{{\text{out}} - {\text{coupling}}}}$ is the optical outcoupling coefficient [17]. At the initial stage of development, research was focused on enhancing the $\eta_{{{\text{radiative}}}}$ of the perovskite emitter layer using compositional, crystal growth, or defect engineering [5, 10, 18]. This has been followed by studies to enhance the $f_{{{\text{balance}}}}$ and $f_{e - h}$ by tuning the device structure, constitutive charge transport, and injection layers [19]. As a result of comprehensive studies on defect passivation and device engineering, $f_{{{\text{balance}}}}$, $f_{e - h}$, and $\eta_{{{\text{radiative}}}}$ have been continuously improved to enhance the internal quantum efficiency of PeLEDs. However, as described by the above-mentioned equation, the EQE is inevitably affected by $f_{{{\text{out}} - {\text{coupling}}}}$. In contrast to research on other factors, relatively few studies have been conducted on improving $f_{{{\text{out}} - {\text{coupling}}}}$.

Similar to the LHE, light outcoupling is governed by the refractive indices of the constitutive materials and their nanostructures. When light travels through heterointerfaces, reflection occurs because of a change in the refractive index of the traveling medium. The refractive index (n) of semiconducting materials can be approximated using the moss relationship as follows [20]:

5 $$n^{4} E_{g} = 95 eV$$

where *E*_g_ is bandgap of the semiconductor. Based on the moss relationship and experimental measurements, the refractive indices of perovskite light emitters range from ~ 1.9 to 2.4 at their corresponding emission wavelengths [21]. When considering the refractive index of a typical organic charge transporting layer (*n* = 1.6–1.8) and glass (~ 1.5), a refractive index difference at the heterointerface and the resulting reflection and trapping of the emitted light are inevitable. Most of the reported high-performance PeLEDs has a planar heterojunction structure, where light trapping via the waveguide mode can be significant. For example, approximately 10%–15% of the emitted light is estimated to be lost by the waveguide mode in PeLED devices, based on an optical simulation (Fig. 1c) [22, 23]. The tuning of the refractive index of the constitutive layer is limited by its effect on the electronic properties of the materials. Therefore, another feasible and effective approach is to design a nanostructure that can minimize the light-trapping loss inside the device and enhance light outcoupling. The PLQY can be enhanced from 20% to 57% through the adoption of a nano/micro structure in the perovskite film and substrate (Fig. 1d) [24]. Therefore, the construction of a well-designed nanostructure may significantly contribute to the light outcoupling efficiency of PeLEDs and thereby considerably enhance the performance of conventional PeLEDs. Furthermore, achieving the high-resolution and well-defined micropatterning of perovskite films and devices is of critical importance to the practical application of PeLEDs in full-color displays and/or micro-LEDs.

## Nanotexturing Processes

To maximize the LHE or light outcoupling of devices in a certain wavelength region, the fabrication of perovskite and/or other functional layers with desired nanopatterning is essential. The nanopatterning of the thin films can be achieved using either top-down or bottom-up approaches. In this section, we summarize nanopatterning methodologies based on top-down and bottom-up approaches.

### Top-Down Approaches

#### Nanoimprinting-Assisted Patterning Methods

Nanoimprinting is a conventional lithography method that uses a soft stamp and is a widely used representative top-down patterning approach for fabricating two-/three-dimensional (2D/3D) multiscale structures on the desired target surface. Polydimethylsiloxane (PDMS) is a thermosetting elastomer that can replicate various structures from the mother-mold; it is typically used as a soft stamp because of its low surface energy and good deformability. The advantage of the nanoimprinting method is that it can be directly patterned on the target surface using a periodic well-defined nanostructure over a large area formed on the surface of the soft stamp. In general, both methods are commonly used to apply nanoimprinting technology to perovskite optoelectrical devices by imprinting on an electron transport inorganic layer, such as a TiO_2_ or ZnO layer, or imprinting on a perovskite active layer [25–35]. In both methods, a gel precursor and solution, which form a functional layer in the device, are deposited on a substrate, and a soft stamp is smoothly imprinted on the surface at a constant pressure using an annealing process to form a nanostructure.

**Fig. 2:**
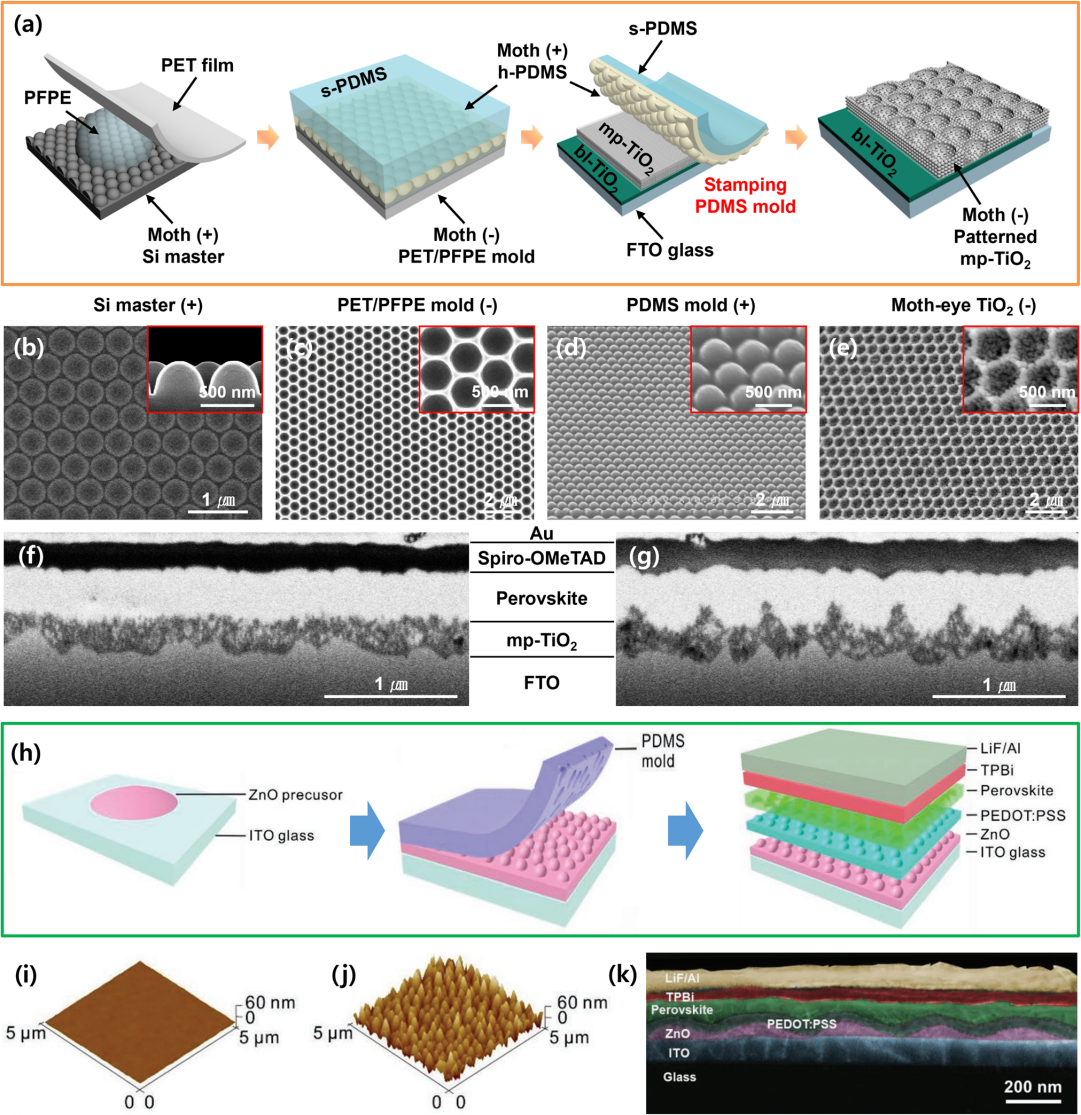
**a** Schematic of the fabrication process of moth-eye-patterned mp-TiO_2_ layers using the PDMS stamping method. **b–e** SEM images showing the results of each process during fabrication. SEM images comparing device morphology fabricated **f** without and **g** with a moth-eye patterned mp-TiO_2_ layer. Reproduced from Ref. [36] with permission from John Wiley and Sons. **h** Schematic of the fabrication process, **i–j** related AFM results, and **k** SEM image of PeLEDs with a nanopatterned ZnO layer. Reproduced from Ref. [38] with permission from John Wiley and Sons

Park et al. contributed to increasing the LHE of PSCs, through research inspired by the function and structure of moth eyes in nature [36]. Moth eyes are composed of numerous nanoprotrusion structures, and they play an important role in reducing the reflectivity of light incident from the outside. Light reflection occurs at the interface between the two different materials. In this case, the larger the difference between the refractive indices of the two materials, the higher the reflectivity. Nanoconical structures, such as moth eyes, serve to reduce the overall reflectivity by gradually reducing the difference in the refractive index for the incident light [36, 37]. In this study, this principle was applied to the mp-TiO_2_ layer of PSCs to increase the amount of light absorbed by the perovskite active layer. First, a PDMS stamp with well-ordered periodic bioinspired nanostructures was replicated from the prepared semipermanent silicon master, as shown in Fig. 2a–e. To obtain an embossed moth-eye-patterned PDMS stamp, a perfluoropolyether (PFPE) engraving mold with a polyethylene terephthalate (PET) film as the substrate was first fabricated from the silicon master. Thereafter, a commercial TiO_2_ paste diluted solution was spin-coated on the device substrate, and the fabricated PDMS stamp was gently covered on the coated surface. After drying the solvent at 125 °C, the PDMS stamp was peeled off smoothly, and the device surface was annealed at 500 °C to obtain a moth-eye TiO_2_ layer. Finally, PSCs embedded with a moth-eye TiO_2_ layer were fabricated. Cross-sectional scanning electron microscopy (SEM) images in Fig. 2f–g reveal the difference between the as-fabricated device and the reference device fabricated with a flat TiO_2_ layer. Tang et al. also used a nanoimprinting method to fabricate nanostructured interfaces between the front electrode and perovskite layer to improve the outcoupling efficiency of PeLEDs (Fig. 2h) [38]. A sol–gel-derived ZnO precursor was spin-coated on ITO glass, and a prepared PDMS stamp with a nanostructure was pressed under conformal pressure and post-annealed on a hot plate at 150 °C. After detaching the PDMS stamp, PEDOT: PSS, CsPbBr_3_ perovskite, TPBi, LiF, and Al were sequentially deposited to complete the PeLED device. The differences in the surface morphologies of the flat and nanostructured ZnO layers on the ITO glass are shown in the atomic force microscopy (AFM) images in Fig. 2i–j. A cross-sectional SEM image of the fabricated device with the patterned ZnO layer is shown in Fig. 2k.

**Fig. 3:**
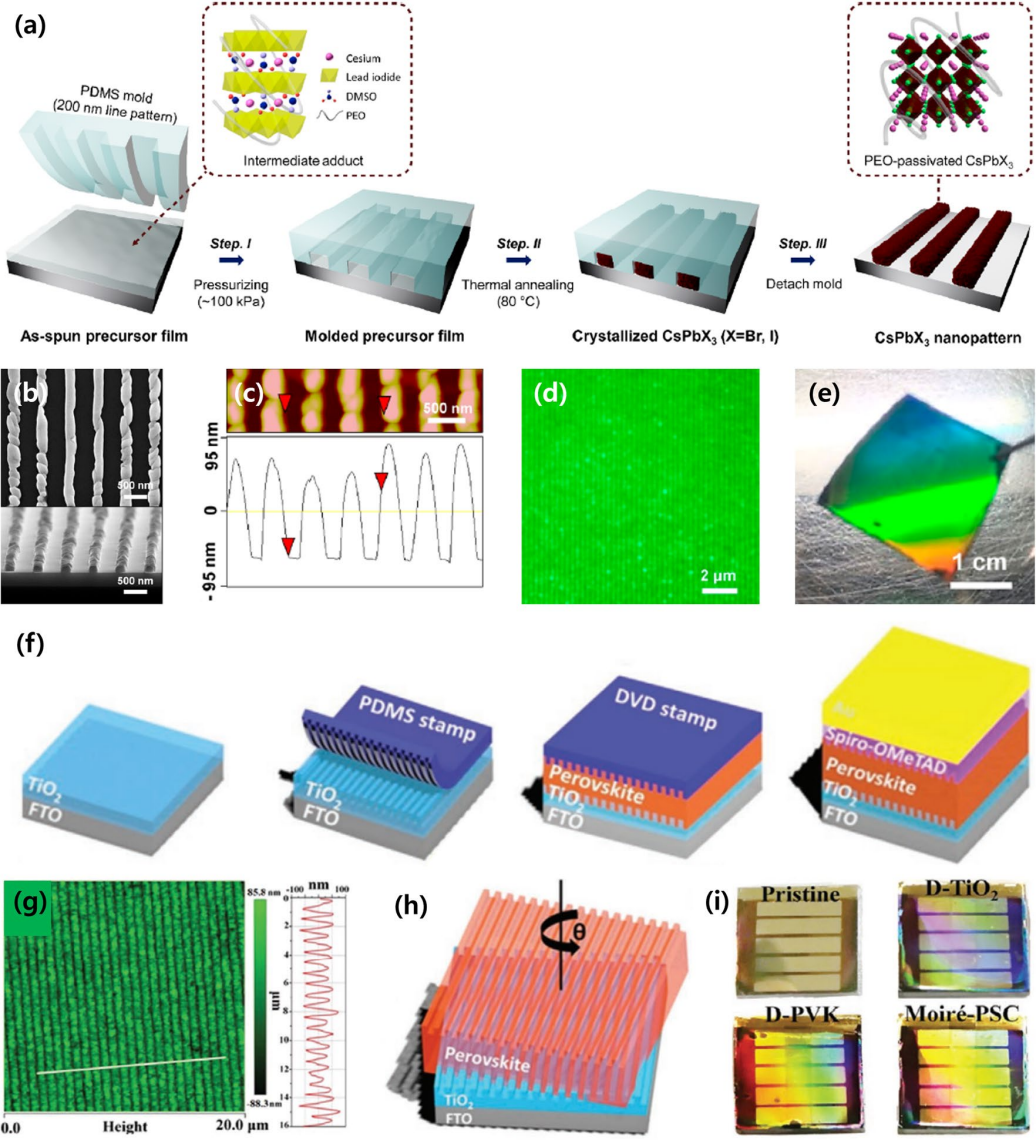
**a** Schematic of the fabrication process to form CsPbX_3_ nanopatterns. **b** SEM image, **c** AFM profile, **d** photoexcited fluorescence image, and **e** digital camera image of the fabricated nanopatterned CsPbBr_3_ device. Reprinted from Ref. [25] with permission from American Chemical Society. **f** Schematic of a perovskite layer with dual diffraction gratings imprinted by a PDMS stamp. **g** AFM image of the grating-structured perovskite film. **h** Schematic presenting the principle of the structural moiré effect. **i** Photographs of the fabricated colorful moiré perovskite solar modules. Reproduced from Ref [26]. with permission from John Wiley and Sons

Recently, Park et al. used the nanoimprinting process to directly crystallize the perovskite layer into the shape of a nanostructure Fig. 3a [25]. To delay the crystallization of the perovskite film during the nanoimprinting process, a soft and moldable inorganic halide perovskite precursor with a polymer additive, poly(ethylene oxide) (PEO), was prepared. First, the polymer additive solution was spin-coated onto a SiO_2_-grown silicon substrate. Thereafter, a prepared PDMS stamp with a 200 nm line pattern was rapidly placed on the substrate in conformal contact. During thermal annealing at 80 °C, CsPbX_3_ crystallizes slowly, and when the PDMS stamp is removed, 200-nm-pitch perovskite nanostructures are formed. The SEM image, AFM profile, photoexcited fluorescence image, and photograph of the fabricated nanopatterned CsPbBr_3_ surface are shown in Fig. 3b–e. Furthermore, Song et al. developed colorful moiré interference nanostructured PSCs using a nanoimprinting process [26]. A moiré interference structure formed by combining two diffraction grating structures was applied to the PSCs; it exhibited an extraordinary light management ability. To demonstrate the structural moiré effect in the photovoltaic device, both the TiO_2_ electron transport layer and perovskite active layer were sequentially patterned to form a device stack using the imprinting method with a nanograting-structured PDMS stamp (Fig. 3f). The TiO_2_ layer was patterned in a manner similar to the above-mentioned nanoimprinting method. Thereafter, a MAPbI_3_ or (FAPbI_3_)_1-*x*_(MAPbBr_3_)_*x*_ precursor solution was spin-coated onto the nanopatterned TiO_2_ surface. After the nanoimprinting process using the PDMS stamp, an AFM image of the fabricated patterned perovskite layer with a grating structure of a period, width, and height of 750, 250, and 100 nm, respectively, is shown in Fig. 3g. To enhance the LHE using the principle of the structural moiré effect, different intersection angles were applied at the grating interface between the TiO_2_ and perovskite layers, as shown in Fig. 3h. This can be easily implemented by rotating and imprinting the PDMS stamp in the sequential nanoimprinting process. The visual images of the fabricated colorful moiré perovskite solar modules are shown in Fig. 3i.

#### Photolithography-Assisted Patterning Methods

**Fig. 4:**
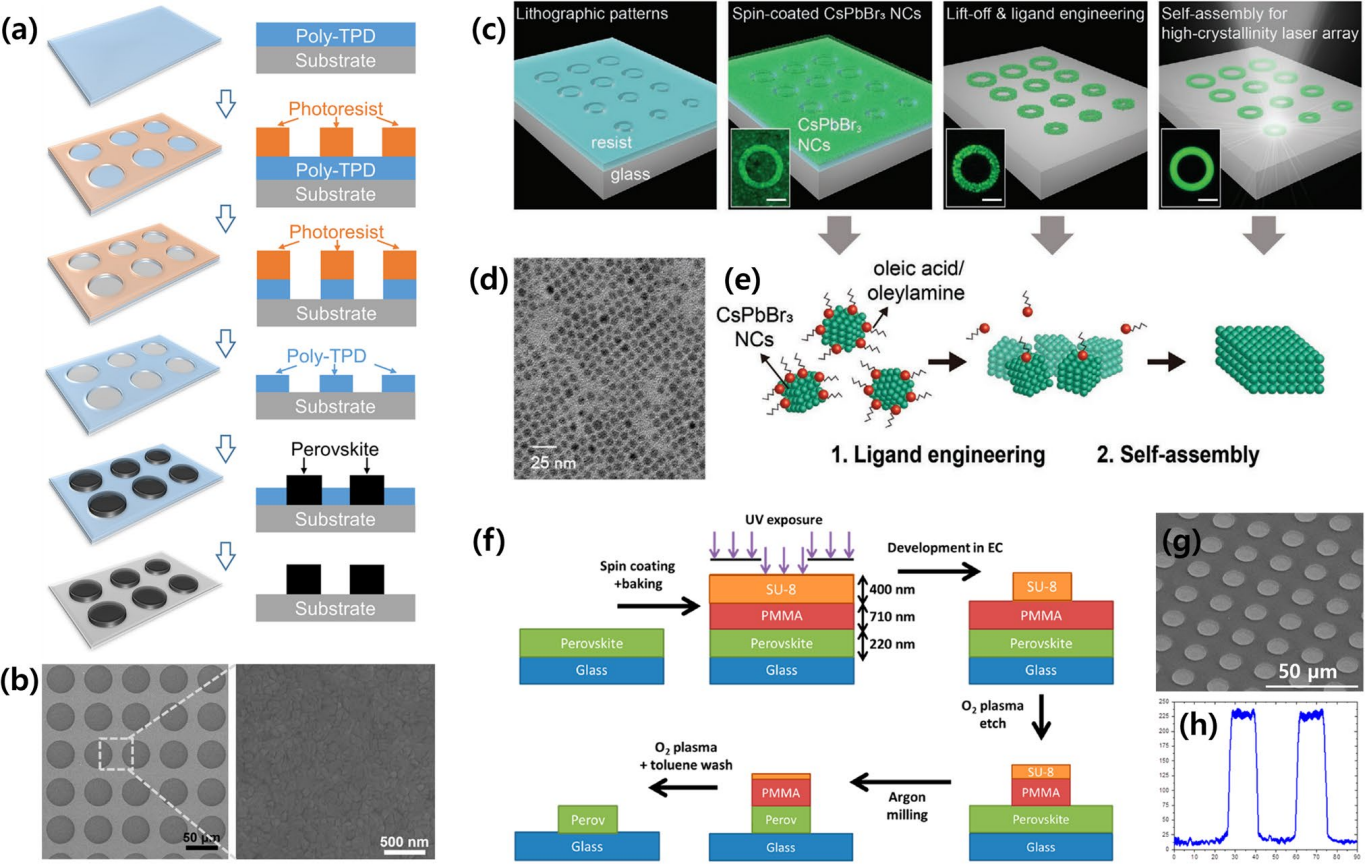
**a** Schematic of the WAP CH_3_NH_3_PbI_3_ patterning process. **b** SEM images of the fabricated CH_3_NH_3_PbI_3_ layer. Reprinted from Ref [39]. with permission from American Chemical Society. **c** Schematic of the self-healing lithographic patterning method to form CsPbBr_3_ NCs. **d** TEM image of the fabricated CsPbBr_3_ NCs. **e** Schematics representing the principles of ligand engineering and the self-assembly process. Reproduced from Ref. [40] with permission from John Wiley and Sons. **f** Schematic of combining the photolithography process and Ar-ion milling approach to form multicolor hybrid (PEA)_2_(MA)_2_Pb_3_Br_10_ perovskite films. **g** SEM image and surface profile of the fabricated (PEA)_2_(MA)_2_Pb_3_Br_10_ patterns. Reprinted from Ref. [41] with permission from American Chemical Society

Photolithography is a representative semiconductor process used to create fine patterns on the surface of a thin film or desired substrate. The photolithography process consists of spin coating a photoresist onto a target surface and irradiating a light source with an appropriate wavelength. The light filtered using a photomask with a geometric pattern imprints the corresponding pattern on the deposited photoresist. Using this mask-shielding method, sophisticated microscale patterns can be fabricated with a high throughput on the substrate [39–50]. Gong et al. developed a wettability-assisted photolithography (WAP) patterning process using a solution of poly(4-butylphenyl-diphenyl-amine) (poly-TPD) in chlorobenzene to fabricate pinhole-free hybrid perovskite films with arbitrarily shaped micropatterns (Fig. 4a) [39]. After the deposition of the poly-TPD layer on the pre-cleaned substrate, a conventional photolithography process was conducted to form a periodic micropatterned photoresist template on the pre-coated poly-TPD layer. Thereafter, chlorobenzene was spin-drop-casted onto the surface to remove the poly-TPD layer and pattern the shape of the photoresist template. After removing the residue from the photoresist template using N, N-dimethylformamide (DMF), the perovskite layer was deposited via a one-step spin-coating method on the combined surface, which was patterned on the exposed hydrophilic substrate because the poly-TPD layer repelled the perovskite precursor. Finally, pinhole-free perovskite patterns with sharp edges were successfully fabricated on the substrate, as shown in Fig. 4b.

Perovskites Nanocrystals (NCs) and quantum dots (QDs) have attracted increasing attention due to their superb properties including quantum confinement effects, size-tunable emission wavelength, high photoluminescence quantum yield, and low-threshold operation [51]. Recently, Delaunay et al. reported a self-healing lithographic patterning technique based on traditional photolithography using CsPbBr_3_ NCs to fabricate advanced single-mode laser arrays (Fig. 4c) [40]. First, a patterned photoresist template was formed on the pre-cleaned glass substrate via a photolithography process. Subsequently, a solution of perovskite CsPbBr_3_ NCs was spin-coated onto the lithographically patterned surfaces. Next, the lift-off process and ligand engineering were simultaneously performed using a flocculation solvent of ethyl acetate with medium polarity, which dissolved the surface ligands present in the NCs and removed the photoresist templates. Thereafter, a self-assembly process using high-polarity methanol vapor was conducted to obtain CsPbBr_3_ NC lithographic patterns with high quality and crystallinity. A transmission electron microscopy (TEM) image of the fabricated CsPbBr_3_ NCs is presented in Fig. 4d, and the principles of ligand engineering and self-assembly are illustrated in Fig. 4e.

Using another method, Samuel et al. patterned multicolor hybrid perovskite films by combining a photolithography process and an Ar-ion milling approach (Fig. 4f) [41]. First, a perovskite precursor solution of (PEA)_2_(MA)_2_Pb_3_Br_10_ was deposited on the prepared glass substrate using the Lewis-base adduct approach. Thereafter, poly(methyl methacrylate) (PMMA) was coated on the surface as a protectant for the perovskite layer. Subsequently, the SU-8 template that acted as an etch mask to duplicate the pattern on the PMMA layer was patterned using a conventional photolithography process. After the O_2_ plasma etching of PMMA to create a shielding barrier, the exposed perovskite film was removed via Ar-ion milling. As a result, microdisk-shaped perovskite patterns were obtained after washing the remaining PMMA and SU-8 masks using additional O_2_ plasma etching and immersing the sample in toluene solution. The SEM images and surface profiles of the patterned perovskite films are shown in Fig. 4g–h.

#### Laser-Assisted Patterning Methods

**Fig. 5:**
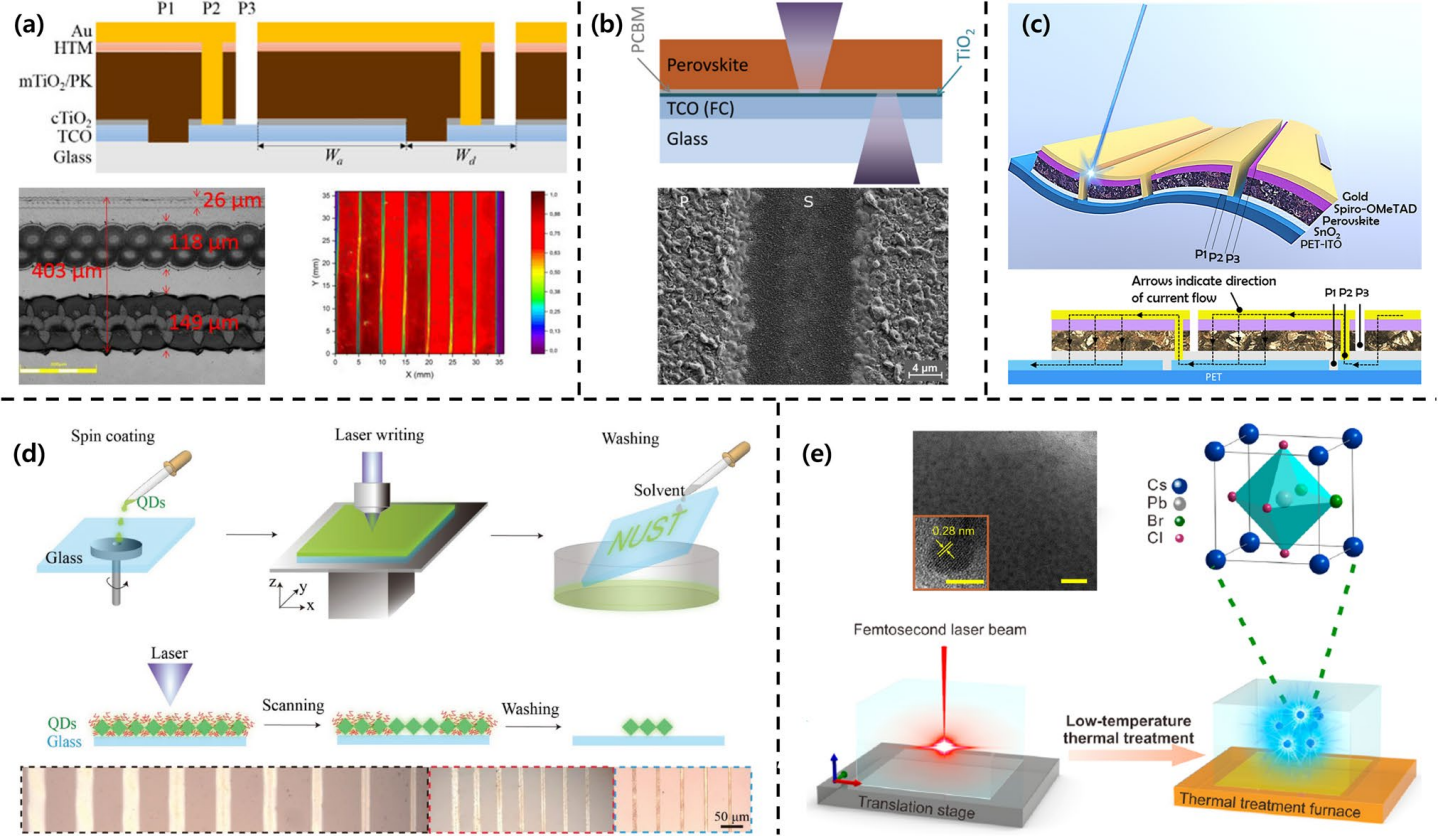
**a** Schematic, SEM image, and LBIC map of the solar minimodule with an area of 14 cm^2^ fabricated using the thin-film laser scribing method. Reproduced from Ref. [52] with permission under a Creative Commons Attribution 3.0 License. **b** Schematic and SEM image of the laser-scribed perovskite films on FTO glass via irradiation using nanosecond and picosecond laser pulses from both sides. Reproduced from Ref. [53]. with permission from Elsevier. **c** Schematics showing laser scribing optimization for flexible perovskite modules. Reproduced from Ref. [54] with permission under a Creative Commons Attribution 4.0 License. **d** Schematic and optical microscopy images of the fabricated perovskite QDs using the LDW method. Reproduced from Ref. [55] with permission from John Wiley and Sons. **e** Schematic and SEM image of blue-emissive metal halide perovskite NCs using the recoverable 3D laser-assisted patterning method. Reprinted from Ref. [56]. with permission from American Chemical Society

Laser scribing or laser direct writing (LDW) methods are facile and fast processes for patterning perovskite materials. These laser-assisted perovskite patterning methods are a type of multiscale structuring techniques that are mask-free and programmable. Laser scribing is a noncontact process using a high-peak-power laser with high accuracy and throughput that scribes small line structures on a substrate coated with organic/inorganic materials [50–57]. Nicolay et al. reported thin-film laser patterning techniques to fabricate high-efficiency perovskite solar devices with an enlarged active area [52]. A fabricated solar minimodule with an area of 14 cm^2^ using the laser scribing method demonstrated a PCE of 16% and a fill factor of 92%. The monolithic interconnection of the fully laser-scribed module was fabricated in three scribing steps: P1, P2, and P3, as shown in Fig. 5a. P1 patterned the conductive layer; P2 connected two electrodes in series, and P3 was used for neighboring cell isolation. The confocal microscope image of the fabricated P1/P2/P3 interconnection line and the light-beam-induced current map of Cs_0.05_(MA_0.17_FA_0.83_)_0.95_Pb(I_0.83_Br_0.17_)_3_ (CsFAMA) devices are shown in Fig. 5a. Zimmer et al. also demonstrated the laser scribing of CH_3_NH_3_PbI_3_ (MAPbI_3_) films on FTO glass using nanosecond and picosecond laser pulses by irradiation from both the front (film) and rear (glass) sides (Fig. 5b) [53]. The laser-scribed area (S) crossing the pristine area (P) can be observed in detail in the SEM image. Furthermore, Brunetti et al. recently reported laser scribing optimization for flexible perovskite modules (FPSMs) with an automatized spray coating of SnO_2_ layers (Fig. 5c) [54]. Laser scribing procedures are significantly more challenging to apply to flexible substrates than to rigid substrates. A nanosecond UV laser with a wavelength of 355 nm was used to obtain the P1, P2, and P3 interconnection lines. Furthermore, they replaced the TiO_2_ film as a conventional electron transport layer with a SnO_2_ layer and confirmed the formation of robust FPSMs using morphological and electrical analyses of laser scribing techniques.

LDW is a mask-free and programmable patterning technology based on the laser–material interaction [55–62]. Zeng et al. proposed a facile and fast patterning process for perovskite QDs using the LDW method (Fig. 5d) [55]. The process consists of three parts: the spin coating of the perovskite precursor, laser irradiation to remove the surfactant around the QDs, and solvent washing of the area with the surfactant. The surfactant near the QDs coated on a glass substrate on the programmed 3D stage was selectively removed in the desired pattern shape using a focused continuous-wave laser with a wavelength of 405 nm. The residual part that was not exposed to the laser was washed with an organic solvent to obtain the desired QD patterns. By adjusting the scanning speed and irradiation energy of the laser irradiated on the substrate, linewidth variation could be realized, as shown in the optical microscopy image. Recently, Dong et al. reported a recoverable 3D laser-assisted patterning method for blue-emissive metal halide perovskite NCs (Fig. 5e) [56]. They achieved a localized crystallization of perovskite NCs in glass by partially irradiating with femtosecond lasers and the subsequent low-temperature thermal treatment of CsPb(Cl/Br)_3_ precursor glass. Femtosecond lasers with a high peak power interacted with a glass matrix containing Cs, Pb, Br, and Cl, which was expected to induce localized ion migration to form NCs. In addition to crystallization, the erasing of CsPb(Cl/Br)_3_ NCs inside the oxide glass was demonstrated using additional laser irradiation and low-temperature thermal treatment.

### Bottom-up Approaches

#### Crystal Growth Engineering of Perovskite Films

**Fig. 6:**
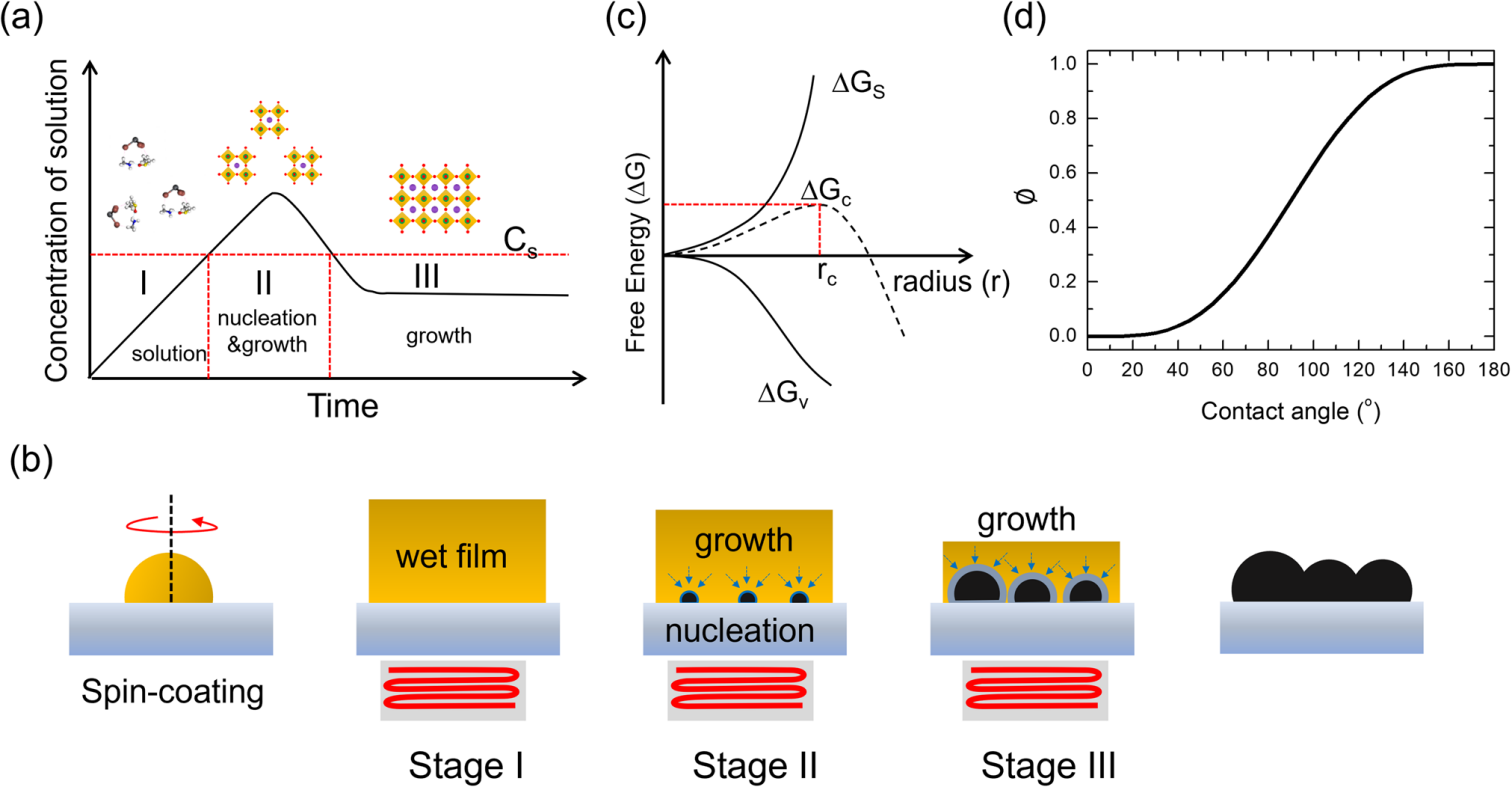
**a** LaMer model for the nucleation and growth of perovskite thin films. C_s_ is the supersaturation concentration of the precursor solution. **b** Schematic of the nucleation and growth of perovskite films at each stage. **c** Free energy diagram of nucleation. ΔG_s_, surface free energy; ΔG_ν_, bulk free energy; ΔG, total free energy, ΔG_c_; critical free energy; r_c_, critical radius of nucleus. **d** Correction term for heterogeneous (⌀) nucleation as a function of the contact angle of the solution on the substrate. Reproduced from Ref. [1] with permission from John Wiley and Sons

The fine control of crystal growth is essential for the patterning of metal halide perovskites. In a typical crystallization process of perovskite films, the La Mer mechanism can be applied, as shown in Fig. 6a [1]. In stage I, the perovskite precursors exist as ions and molecules in the precursor solution until the concentration of the solution increases to a supersaturation level (*C*_s_) owing to solvent evaporation. As the concentration reaches *C*_s_, perovskite crystal nuclei start to form and grow with the supply of solute owing to a diffusion process (stage II). Nucleation continues until the concentration of the solution decreases below *C*_s_, as the consumption of the solutes is faster than the evaporation of the solutes in stage III. In stage III, nucleation does not occur, and only the growth of the formed nuclei progresses until the precursor solution is depleted. The progress of the stages during the typical spin-coating process of perovskite thin films is depicted in Fig. 6b. To achieve well-defined patterns using bottom-up approaches, it is necessary to understand the nucleation and growth kinetics during crystallization processes.

The first step of patterning via a bottom-up approach is to induce the nucleation of the perovskite at the desired positions. To control nucleation, an understanding of the factors affecting the nucleation kinetics is essential. Based on the classical nucleation theory (Fig. 6c), the homogeneous nucleation rate is governed by the Arrhenius-type equation as follows [1, 63]:

6 $$\frac{{{\text{d}}N}}{{{\text{d}}t}} = A{\text{exp}}\left( {\frac{{\Delta G_{c}^{{{\text{homo}}}} }}{{k_{B} T}}} \right)$$

where *t* is the time; N is the number of nuclei; $\Delta G_{c}^{{{\text{homo}}}}$ is the critical free energy for homogeneous nucleation; *A* is the pre-exponential factor; $k_{B}$ is the Boltzmann constant; and T is the temperature. Therefore, localized nucleation can be achieved by controlling the $\Delta G_{c}^{{{\text{homo}}}}$ or *T* at the desired position. The above equation can be converted into the following equation using the relationship between $\Delta G_{c}^{{{\text{homo}}}}$ and surface energy $\gamma$, molar volume $v$, and supersaturation of solution $S$ as follows:

7 $$\frac{{{\text{d}}N}}{{{\text{d}}t}} = A{\text{exp}}\left( { - \frac{{16\pi \gamma^{3} v^{2} }}{{3k_{B}^{3} T^{3} \left( {{\text{ln}}S} \right)^{2} }}} \right)$$

Among the variables, $\gamma$ and $v$ are material-specific factors, whereas *T* and S can be controlled by process engineering. Furthermore, it is possible to induce localized nucleation via heterogeneous nucleation at the targeted position, whose activation energy is given by:

8 $$\Delta G_{c}^{{{\text{hetero}}}} = \emptyset \Delta G_{c}^{{{\text{homo}}}}$$

9 $$\emptyset = \frac{{\left( {2 + {\text{cos}}\,\theta } \right)\left( {1 - {\text{cos}}\,\theta } \right)^{2} }}{4}$$

where $\theta$ denotes the contact angle of the solution. Therefore, tuning the contact angle of the solution at the localized position would be another possible approach to control the nucleation of the perovskite (the contact angle-dependent $\emptyset$ value is illustrated in Fig. 6d). When the nuclei are formed, controlling their growth is important to achieve the desired microscopic morphology. Furthermore, defining the position of nucleation using physical templates is feasible. In the following section, we review previous attempts to generate halide perovskite patterns using bottom-up approaches.

#### Surface Dewetting-Assisted Methods

**Fig. 7:**
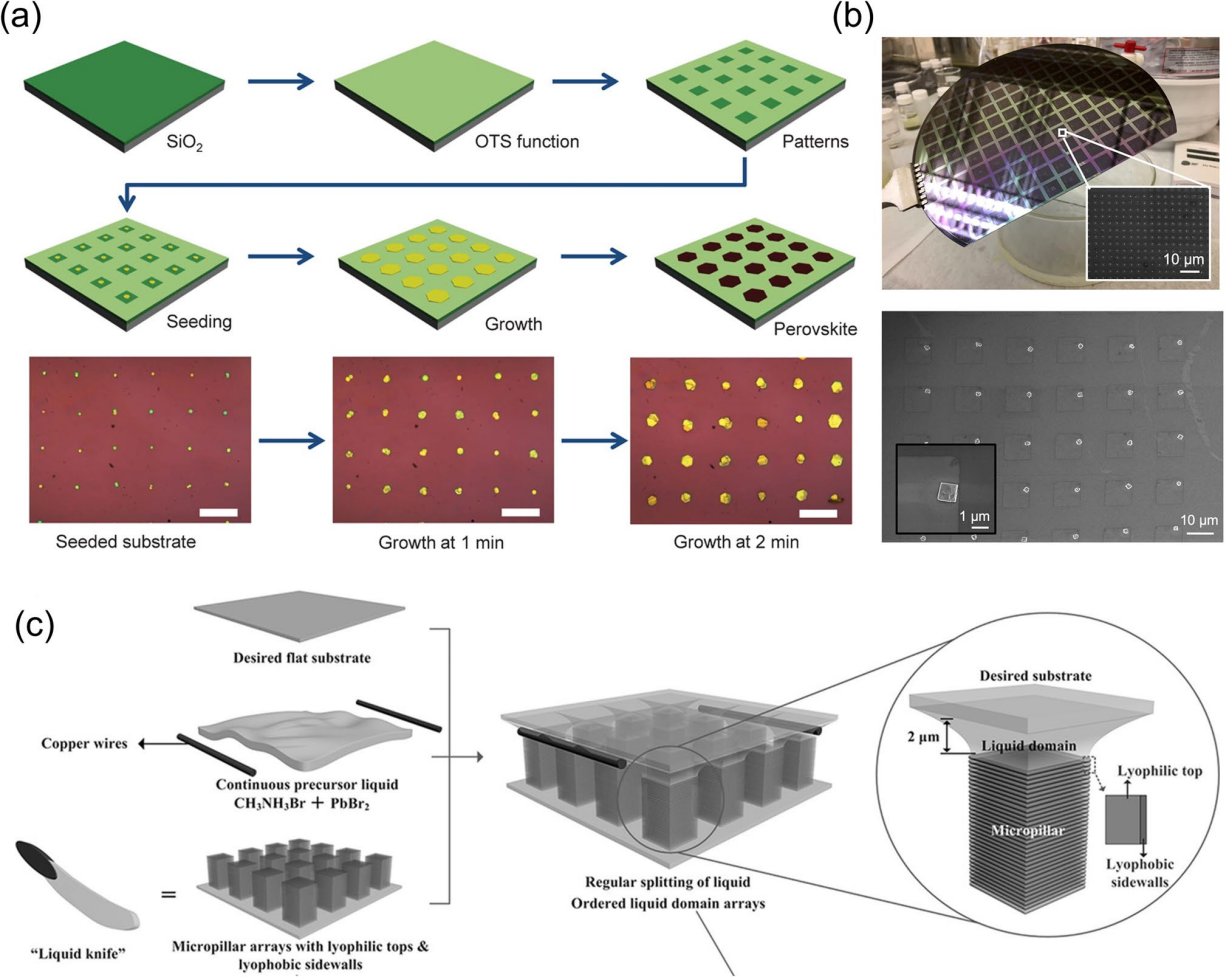
**a** Schematic of the patterned growth of regular arrays of perovskite microplate crystals on a wettability-controlled SiO_2_/Si substrate. Reproduced from Ref. [65]. with permission from American Association for the Advancement of Science. **b** Photo of a 6 in wafer coated with a patterned CsPbBr_3_ microcrystal array. Reproduced from Ref. [66] with permission from American Chemical Society. **c** Schematic of the surface-induced growth of CH_3_NH_3_PbBr_3_ perovskite patterns. Desired flat glass wafer, an approximately 10 μL droplet of perovskite precursor liquid consisting of CH_3_NH_3_Br, PbBr_2_, and DMF, and a pillar-structured “liquid knife” were integrated as a sandwich-shaped reaction system. Reproduced from Ref. [67] with permission from John Wiley and Sons

Based on the above equation for heterogeneous nucleation, the nucleation of a perovskite from a hydrophilic perovskite precursor solution is suppressed on a hydrophobic substrate surface [64]. When the hydrophobicity is sufficiently high, the wetting of the precursor solution is minimal, thereby naturally preventing the nucleation of the perovskite in the hydrophobic region. Wang et al. utilized this strategy by adopting a patterned substrate for the demonstration of perovskite microplate crystal arrays (Fig. 7a) [65]. A hydrophobic self-assembled monolayer of (octadecyl) trichlorosilane (OTS) was used to fabricate a hydrophobic surface region to prevent the wetting of the lead halide precursor solution. The aqueous PbI_2_ solution treatment resulted in the selective formation of PbI_2_ nuclei at the periodic hydrophilic regions, where the OTS was removed via photo- or e-beam lithography followed by oxygen plasma treatment. The conversion of the grown PbI_2_ plates using a CH_3_NH_3_I (MAI) gas treatment enabled the formation of periodic microplate arrays of MAPbI_3_. Lin et al. adopted a similar approach to fabricate all-inorganic cesium lead halide perovskite arrays [66]. Cesium halide was first deposited in the hydrophilic region of the substrate defined by the OTS, which was then converted to cesium lead halide perovskite single crystals via the chemical vapor transport process to deposit lead halides. During the deposition of cesium halide, the wettability of the hydrophilic region was controlled by tuning the density of the additional functional layer (methyltrichlorosilane, MTS) to enable the formation of single nuclei. The wafer-scale fabrication of a single-crystal perovskite array has been successfully demonstrated (Fig. 7b). Instead of controlling the wettability of the substrate, Feng et al. utilized a specially designed liquid knife to generate a liquid domain on the substrate (Fig. 7c) [67]. Contacting micropillar arrays with controlled lyophilic properties formed using a precursor solution enabled the generation of a liquid domain whose pattern is defined by the periodicity of the micropillar. The evaporation of the solvent from the liquid domain enabled the generation of single-crystal perovskite arrays on the substrate.

#### Localized Heating-Assisted Methods

**Fig. 8:**
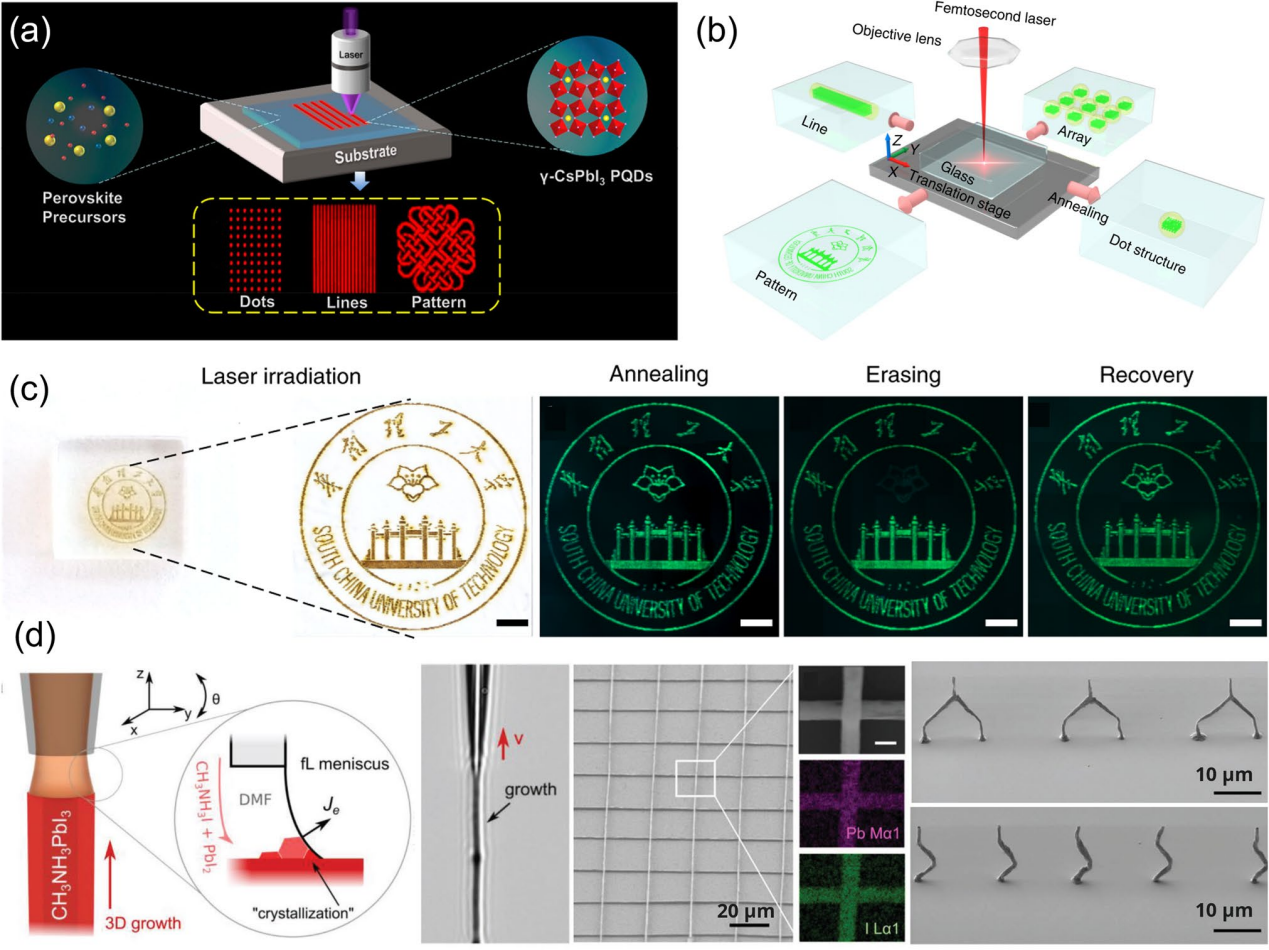
**a** Schematic of the process of the direct laser writing of γ-CsPbI_3_ perovskite QDs. Reproduced from Ref. [59]. with permission from American Chemical Society. **b** Schematic of the femtosecond laser writing system for sample fabrication and **c** demonstration of reversible CsPbBr_3_ QD 2D patterns and 3D structures (scale bars are 500 μm). Reproduced from Ref. [57] with permission from Springer Nature. **d** Schematic showing the meniscus-guided 3D printing of organic–inorganic metal halide perovskites (left), and 3D perovskite architectures printed using this method (right). Reproduced from Ref. [69]. with permission from John Wiley and Sons

Based on the above-mentioned equation, it is possible to induce localized nucleation by controlling the supersaturation of the precursor solution. The halide perovskites demonstrate an inverse dependence of the temperature on the solubility (i.e., the solubility of the perovskite decreases as temperature increases) under specific solvent compositions [23]. As a result, the localized heating of the precursor solution with such solvents can induce the nucleation of the perovskite. Chou et al. utilized this property to induce the selective nucleation of the perovskite on a substrate [68]. The localized heating of the substrate using laser irradiation reduced the solubility of the perovskite in the precursor solution, leading to the formation of nuclei in the irradiated region of the substrate, which subsequently grew into chunks of microcrystals. Zhan et al. also utilized the laser writing approach to fabricate patterned perovskite QDs embedded in a polymer matrix (Fig. 8a) [59]. A composite of perovskite precursor and PMMA was first deposited by spin coating the DMF solution, to which a 405 nm laser was irradiated to remove residual solvent and induce the nucleation and growth of the perovskite QDs. The highly luminescent (PLQY > 90%) CsPbI_3_ QD patterns have a minimum line width as low as 900 nm. Huang et al. designed an oxide glass matrix containing cesium, lead, and bromine to achieve the 3D laser printing of perovskite QDs (Fig. 8b) [57]. The in situ formation of highly luminescent CsPbBr_3_ QDs was achieved via irradiation with a femtosecond pulsed laser. The generated pattern could be erased via further femtosecond laser irradiation. Writing and erasing could be repeated for several cycles (Fig. 8c).

#### Additive Printing-Assisted Methods

Among the various approaches, generating patterns using the additive printing of halide perovskites is probably the most challenging because of the difficulty in controlling the nucleation and growth of crystals. Chen et al. demonstrated the nanoprecision 3D printing of a perovskite based on guiding evaporation-induced crystallization in midair using a femtoliter ink meniscus formed on a nanopipette (Fig. 8c) [69]. Stretching the ink meniscus with a varying pulling speed enabled the on-demand control of the diameter and hollowness of the perovskite nanowire. Meanwhile, varying the pulling direction enabled the realization of 3D architectures with programmable shapes and positions.

Song et al. utilized the direct writing of perovskite ink using inkjet printing to fabricate perovskite single-crystalline microplate arrays [70]. The perovskite precursor ink was printed on the desired position of the substrate, which was subsequently crystallized into a single-crystalline microplate with solvent evaporation. They performed a systematic investigation of the effects of the adhesive force, substrate temperature, and ink droplet volume on the size and morphology of the formed microcrystals. Lin et al. utilized a nanoporous anodic aluminum oxide (AAO) as a template to form perovskite nanowires using the inkjet printing method [71]. The MAPbX_3_ (X = I, Br, Cl) perovskite inks are printed on the AAO template with vacuum pumping to promote infiltration of the ink. The formed perovskite nanowires demonstrated random lasing capability with improved operational and environmental stability owing to passivation effect provided by the compact spatial confinement of the AAO combined with a poly(methyl methacrylate) (PMMA) sealing process. Patterned single-crystalline microplate arrays were successfully fabricated. Gu et al. utilized a similar approach to generate an array of seed crystals for the growth of millimeter-sized perovskite single-crystal films with controlled thickness [72]. Precursor ink was printed at a controlled position on the substrate to generate patterned perovskite seed crystals. The generated seed crystals inhibited random nucleation and triggered the growth of single-crystal films during the subsequent spatial confinement growth stage. As a result, patterned single-crystalline films were successfully formed, which could be transferred onto the target substrates. The direct printing of a perovskite precursor solution on a polymer substrate has also been demonstrated by Li et al. [73] and Shi et al. [74]. It was suggested that the heated polymer substrate was partially dissolved or swelled by the printed ink on it and formed highly luminescent (PLQY up to 80%) perovskite microcrystals after solvent evaporation [74]. Various polymer films were adopted to support the versatility of the approach. Compared to the approach that facilitates the in situ crystallization of the film during printing, the inkjet printing of pre-synthesized perovskite NCs is relatively easier to control. As a result, several studies have been conducted to demonstrate microscale NC patterns using this approach [75–81]. To achieve better printability and film-forming ability, the formulation of the ink solvents is a crucial factor. Recently, Wei et al. demonstrated that the use of a ternary-solvent-ink strategy enables the control of solvent evaporation and the surface tension of the ink to form gradient volatilization and accelerate the evaporation flow, thereby prolonging the Marangoni flows and significantly suppressing the coffee-ring effect for the printed perovskite QD thin films [80].

#### Control of Film Stress

**Fig. 9:**
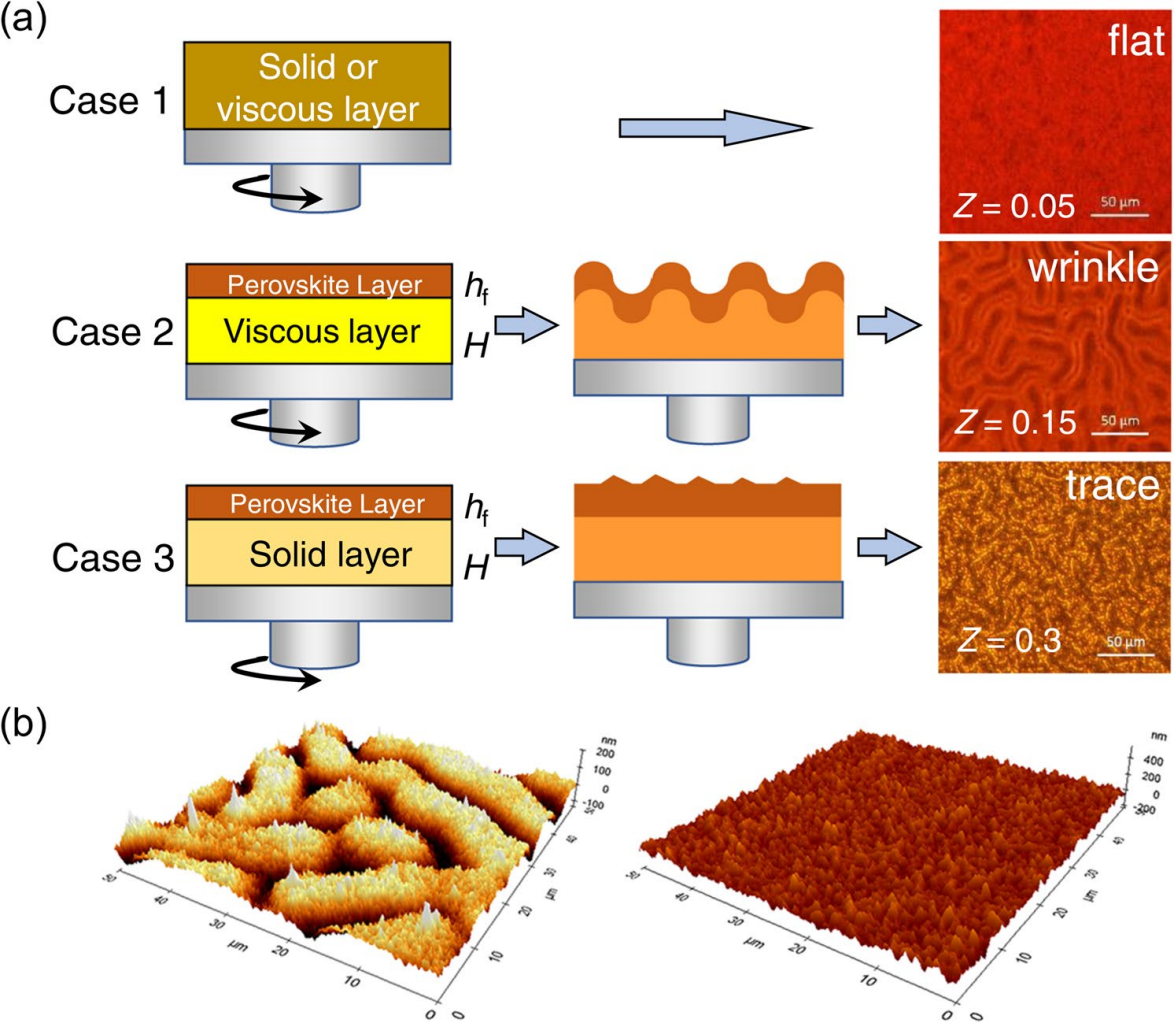
**a** Intermediate-layer structure for the flat surface (case 1), wrinkled structure (case 2), and coarse surface with a trace of wrinkles (case 3). Bilayer model with a solid upper layer with a thickness of h_f_ was proposed to explain the wrinkling process. Reproduced from Ref. [16]. with permission from Springer Nature. **b** AFM images showing the perovskite top surface morphology formed via the antisolvent method (left) and interdiffusion method (right). Reproduced from Ref. [79]. with permission from American Chemical Society

The microstructure of the film can also be affected by evolving film stress during the fabrication process. In-plane stress, either tensile or compressive, can develop in films during fabrication. For instance, during the formation of intermediate adduct phases via antisolvent dripping, the spinning wet film can be partially crystallized from the top surface, where the dropped antisolvent considerably reduces the solubility of the perovskite precursors [78]. The proportion of the crystallized film depends on the miscibility of the antisolvent with the perovskite precursor solution. Consequently, a bilayer consisting of an elastic perovskite top layer and viscoelastic amorphous bottom layer can be formed. In this case, a compressive stress is applied to the viscoelastic bottom layer, which can be dissipated by the formation of a wrinkled structure, as demonstrated by Kim et al. (Fig. 9a) [16]. The wavelength and amplitude of the formed wrinkle are dependent on the composition of the perovskite and the properties of the antisolvent because the thicknesses and mechanical properties of the constitutive layers depend on the aforementioned factors. Meanwhile, in-plane stress can also be produced owing to the difference in the mechanical properties of the coated film and substrate. For example, Bush et al. observed a compressive stress of 21.6 $\pm$ 3.0 MPa in the film immediately after the dripping of chlorobenzene antisolvent, resulting in a wrinkled structure in the film (Fig. 9b) [79]. During the subsequent annealing and cooling processes, the compressive stress can be relaxed, and isotropic tensile stress can be developed owing to the higher coefficient of thermal expansion (CTE) of perovskite (50–160 ${\mu K}^{ - 1}$) than that of the glass substrate (2.6–10 ${\mu K}^{ - 1}$). This marginally affects the film morphology because of the relatively high rigidity of the crystallized film. Contrary to that observed in the one-step process, textured morphology was not observed in the two-step process, where the film remained in biaxial tension during the entire fabrication process. These results indicate that the microscopic texture of the film can be controlled by modulating the fabrication process of the film with an identical perovskite composition.

## Summary and Perspectives

In summary, we have provided an overview of nanopatterning/texturing technologies for perovskites to achieve a high device performance and categorized them into top-down and bottom-up approaches. In the top-down approaches, nanoimprinting-assisted methods are widely used to fabricate various patterns onto perovskite layers using PDMS molds. Additionally, photolithography and laser-assisted methods have the advantages of being able to precisely texture the inner layers of perovskite devices rapidly and simply. Meanwhile, the bottom-up approaches focus on the fine control of perovskite crystal growth starting at the atomic or molecular level. The principles of surface dewetting, localized heating, additive printing, and film stress control methods have been adopted to produce the desired patterns during the crystallization process of perovskite films. The introduced patterning methods can endow perovskite optoelectrical devices with two main advantages: (1) LHE and (2) light outcoupling efficiency. These improvements in nanopatterned-perovskite-based optoelectronic applications result from structural effects, such as antireflection effects, light trapping, and light-scattering mechanisms.

Although significant advancements have been achieved in perovskite nanopatterning for various applications, several issues remain to be addressed as follows: (1) the most representative and frequently employed nanoimprinting-assisted methods are highly dependent on PDMS molds. PDMS easily swells the solvent, which can cause inevitable damage to the perovskite layer [80]. Furthermore, it is difficult to precisely form nanostructures under 500 nm owing to the high viscosity of the PDMS precursor [81]. (2) Limited photoresist or developer is used in photolithography-assisted patterning methods to fabricate high resolution nanopatterns. In addition, the harsh process of heat annealing, etching, and lift-off methods may cause damage to the underlying layers [82, 83]. (3) In laser-assisted nanopatterned methods, the higher the laser power, the more precise the pattern can be fabricated, but it may damage the perovskite layer (e.g. volatilization of organic species) [84]. (4) Meanwhile, bottom-up approaches have complicated procedures, low process speed, and poor film uniformity, and these factors significantly limit the realization of high resolution, large-area fabrication, and mass production [82, 85]. Therefore, the most important issues to be addressed for the further growth of patterning technology for perovskites are related to process reproducibility, device durability, stability, and low processing costs.

Because the various functions afforded by the use of nanostructures clearly improve the performance of perovskite-related devices, we need to continuously develop novel patterning methods. (1) Using hybrid methods of top-down structuring and bottom-up crystal growth, we can obtain more controllable nanopatterns and crystallinity with few defects, and environmental durability [85, 86]. (2) The efficiency of PSCs and PeLEDs can be more increased by enhancing light harvesting and light outcoupling ability by patterning of transparent electrode or forming anti-reflective layer on the substrate using nanoparticles [87, 88]. Comprehensive optical design of all the constitutive layers should be conducted for effective utilization of the light. (3) Electrospinning/spraying perovskite fibers can be another alternative for fabricating flexible and large-scale 3D perovskite applications as it can improve the mechanical stability of the perovskite layer [89, 90]. (4) Replacing PDMS molds to chemically stable polymers such as PFPE can be explored to develop high-performance, more precise nanopatterned perovskite-based optoelectronic applications [80]. 5) High efficiency solar cells (> 30%) with desirable light management can also be accomplished through the conformal deposition of perovskite layers on textured substrate, e.g., textured silicon cells. Development of coating technologies for conformal coating of the perovskite layer on such substrate is another important topic in PSC based tandem devices [91–94].

Moreover, the effects of such patterning strategies should be discussed in terms of long-term stability since it is another critical aspect of the most concerned issues for commercialization of perovskite devices [95]. The patterning of perovskite layer inevitably increases film surface area, which can increase surface defect density and promote ion migration. Thus, effective passivation of the surface defects should be accompanied. As the enlarged interface between perovskite and charge transporting layer can facilitate the charge extraction/collection in the PSC and PeLED devices, it can further contribute to the device efficiency and stability. Although using a cover glass with underlying polymeric sealant is considered the most effective encapsulation method to prevent moisture penetration into perovskite devices that may cause chemical degradation of the material, it has some limitations, such as sealant thermal/UV curing process, and is difficult to use on flexible/stretchable/thin substrate. From this point of view, patterned polymer films with liquid repellent properties have gradually begun to receive attention as an alternative to secure the stability of perovskite devices [96, 97]. The thin polymer film with multiscale structures on the surface originally block the contact of liquid droplets, which replace the role of cover glass, and may have additional properties including flexibility, stretchability, and reusability. Overall, we are confident that the advancements in the nanopatterning technology will significantly influence the future technological development of perovskite optoelectrical devices.
